# *Rickettsia felis*: A Review of Transmission Mechanisms of an Emerging Pathogen

**DOI:** 10.3390/tropicalmed2040064

**Published:** 2017-12-19

**Authors:** Kelsey P. Legendre, Kevin R. Macaluso

**Affiliations:** Department of Pathobiological Sciences, School of Veterinary Medicine, Louisiana State University, Baton Rouge, LA 70803, USA; kmacal2@lsu.edu

**Keywords:** *Rickettsia felis*, transmission, fleas

## Abstract

*Rickettsia felis* is an emerging pathogen of the transitional group of *Rickettsia* species and an important cause of febrile illness in Africa. Since the organism’s original discovery in the early 1990s, much research has been directed towards elucidating transmission mechanisms within the primary host and reservoir, the cat flea (*Ctenocephalides felis*). Several mechanisms for vertical and horizontal transmission within this vector have been thoroughly described, as well as transmission to other arthropod vectors, including other species of fleas. However, while a growing number of human cases of flea-borne spotted fever are being reported throughout the world, a definitive transmission mechanism from arthropod host to vertebrate host resulting in disease has not been found. Several possible mechanisms, including bite of infected arthropods and association with infectious arthropod feces, are currently being investigated.

## 1. Introduction

**Figure 1:**
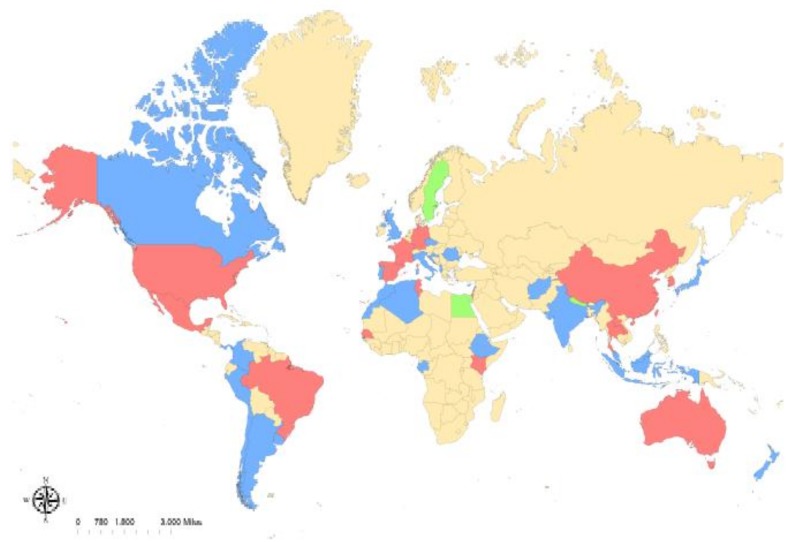
Reported global distribution of *Rickettsia felis*. *R. felis*-positive arthropods have been reported in (blue): Afghanistan, Albania, Algeria, Argentina, Australia, Canada, Chile, Colombia, Croatia, Cyprus, the Czech Republic, the Democratic Republic of Congo, Ethiopia, Gabon, India, Indonesia, Israel, Italy, Ivory Coast, Japan, Lebanon, Malaysia, Morocco, Panama, Peru, Portugal, Romania, the United Kingdom, and Uruguay. Along with infected arthropods, human cases of *R. felis* have been reported in (red): Australia, Brazil, China, France, Germany, Kenya, Laos, Mexico, New Zealand, South Korea, Spain, Taiwan, Thailand, Tunisia, and the United States. Human cases without detection of infected arthropods have been reported in (green): Egypt, Nepal, and Sweden.

*Rickettsia felis* is an obligate intracellular bacterium of the transitional group of *Rickettsia* species, and is the causative agent of emerging flea-borne spotted fever [1]. This organism was first associated with human disease in a patient from Texas in 1994 [2], and human cases have since been reported on every continent except for Antarctica [3]. The widespread nature of the disease is likely secondary to the believed primary vector and reservoir host, the cat flea (*Ctenocephalides felis*), which shares a similar pervasive range (Figure 1). While much work has been done to investigate the spread of *R. felis* between cat fleas and to vertebrate hosts, a definitive transmission mechanism that produces a rickettsemic host with clinical signs that mimic the human disease has yet to be found.

Recent studies have associated *R. felis* to infection and febrile illness in Africa, with up to 15% of patients with fever of unknown origin having detectable levels of *R. felis* in their blood via PCR analysis [4]. There have also been recent outbreaks of flea-borne rickettsiosis in the United States, including Texas, California, and Hawaii, where data has shown *R. felis* to be more prevalent in arthropods and mammals via PCR analysis in the area than *R. typhi* (the etiologic agent of murine typhus) [5,6,7]. Murine typhus is another flea-borne rickettsioses, of the typhus group *Rickettsia* spp., causing a disease clinically indistinguishable from *R. felis*. This agent also has endemic foci in southern California and south Texas [4]. Given the similarities in clinical presentations and location of outbreaks, it is evident how many of these cases could be confused with each other, as well as other similar rickettsial diseases. The advent of more sophisticated diagnostic techniques has aided in the distinction of some of these cases [8], allowing for a clearer clinical picture of flea-borne spotted fever.

## 2. Background

Rickettsiosis is caused by bacteria of the genus *Rickettsia*, which includes the spotted fever group (SFG), typhus group (TG) and, a more recent classification, transitional group (TRG) [9]. *Rickettsia* spp. are most commonly divided into the SFG or TG based on their vector of transmission, antigenic characteristics, optimal growth temperatures, percent G + C DNA contents, and clinical features [10]. Bacteria associated with the SFG are usually transmitted to vertebrates via the bites of hard ticks, while members of the TG are predominantly transmitted by contamination of mucous membranes, conjunctivae, and/or open wounds with the infectious feces of lice and fleas [11]. *Rickettsia felis* was originally characterized as a typhus-like *Rickettsia* due to the fact that the first human case was originally misdiagnosed as murine typhus and the organism was initially isolated from a laboratory flea colony. Additionally, early analysis of the 17 kDA and citrate synthase genes of *R. felis* supported a TG classification [12]. However, later analysis revealed the presence of the *omp*A gene and a 17-kDA gene having more similarity to the SFG rather than TG [1]. There has been some debate in the literature regarding whether to classify *R. felis* as TRG *Rickettsia* sp. or a SFG variant. While some agree with the creation of a third group of *Rickettsia* spp. (TRG), others still classify *R. felis* as a SFG *Rickettsia* sp. The latter classification for *R. felis* as a SFG-variant coincides with other organisms that are also transmitted by arthropods other than ticks (e.g., *R. felis*-like organisms and *R. hoogstraalii*) [12]. The difficulty in even classifying this emerging pathogen helps display part of the obstacles that many have had in distinguishing *R. felis* from other related bacteria in both clinical and laboratory settings.

## 3. Clinical Disease

The clinical manifestation of several rickettsioses, specifically *R. felis* and *R. typhi*, have many similarities, including headache, chills, fever, myalgia, and malaise, with a large number of patients presenting with a maculopapular rash [4]. Few cases have presented with an ‘eschar’, which is a single, crusted, cutaneous lesion surrounded by inflammation, thought to represent the site of inoculation via an arthropod [13]. It has been reported that the percentage of patients that present with rashes and eschars (75% and 13%, respectively) is higher in cases of *R. felis* compared to *R. typhi* [4]. Rarely, *R. felis* has also been associated with neurologic signs (including a polyneuropathy-like syndrome and subacute meningitis), pneumonia, and gastrointestinal symptoms [14]. To date, there have been no reports of *R. felis* causing more serious complications or death [1]. However, the similarity of flea-borne spotted fever symptoms to *R. typhi* and other vector-borne diseases, as well as the lack of specific diagnostics, has potentially led to an underdiagnosis of *R. felis* in many human cases.

As stated previously, *R. felis* has been reported as an emerging cause of fever of unknown origin in Africa. However, given the fact that *R. felis* has also been detected in skin swabs from afebrile patients in Africa [15], it has been suggested that the organism is ubiquitous in the area and its true pathogenicity has been questioned [16]. To explain the variable presentations, it has been proposed that patients in Africa exhibit a more chronic form of the disease, with disease-free intervals interspersed with periods of relapse (similar to malaria, which shares a common epidemiology with *R. felis* in certain areas of Africa) [14]. Adding to the perplexing nature of *R. felis* in Africa, several studies performed in areas of outbreaks have surprisingly not been able to detect *R. felis* in local cat fleas [14]. However, the organism was able to be detected in multiple species of mosquitoes, including several *Anopheles* spp. Survey studies in the area have shown a correlation between the locations of *R. felis*-infected mosquitoes and human infections [17], as well as a correlation between infected mosquitoes and the prevalence of *R. felis* in ape feces [18], suggesting a potential role for alternate hosts in the disease ecology in Africa.

One of the strongest correlations between *R. felis* and human disease in Africa came from a recent case study that described a vesicular fever in an 8-month-old girl in Senegal [15]. The patient erupted in vesicles and ulcers over her entire body and presented with a fever. Swabs of the cutaneous lesions were performed, which were found to be qPCR-positive for *R. felis*, although qPCR results were negative in the blood samples. Additionally, serum samples collected from time points prior to infection, as well 40-days post-presentation, were negative for *R. felis* antibodies via immunofluorescence assay (IFA) and Western blot analysis. Given the lack of seroconversion in the patient, this was described as a primary infection of *R. felis* causing the clinical cutaneous presentation. Researchers proposed the term ‘yaaf’, the Senegalese word for vesicle, to identify the clinical entity [15]. Another case of a primary infection was described previously in the Yucatan, with similar lesions, suggesting the specific cutaneous lesions may be pathognomonic for *R. felis* [19]. The inability to isolate *R. felis* from blood, even in acutely ill patients, has been thought to preclude a definitive link between the organism and disease. However, these recent case studies have shown that there is a possible alternative route to disease that does not include circulating blood-borne rickettsial organisms.

## 4. Transmission in Arthropods

To date, thirty-nine species of arthropods have been associated with *R. felis*, including several species of fleas, ticks, lice, and mosquitoes [20]. However, the cat flea (*Ctenocephalides felis*) has been shown to serve as not only the primary vector, but seemingly the reservoir of *R. felis* in the environment as well [3,21]. The maintenance of *R. felis* within laboratory colonies of cat fleas has been extensively studied, and was originally attributed mainly to vertical transmission, or the transmission of pathogen from parent to offspring [12,22]. Strong evidence for this mechanism was given when *R. felis* was found to be present in both male and female cat flea reproductive tissue, including the ovaries and epithelial sheath of the testes [23]. *Rickettsia felis* was first shown to undergo transovarial transmission, with detection of *R. felis* in freshly-deposited cat flea eggs [12], followed by the exhibition of *R. felis* in newly-emerged unfed adult cat fleas, demonstrating transstadial transmission [24]. However, vertical transmission of *R. felis* to the progeny of cat fleas has reported to be highly variable, with several studies demonstrating the inability of cat fleas to maintain vertical transmission of *R. felis* when exposed as adults [22,25,26]. While observed variability in vertical maintenance is likely a laboratory artifact, this lack of transmission to progeny during infection bioassays suggests that alternate mechanisms to introduce and maintain *R. felis* in vector populations likely exist.

The ability to undergo frequent horizontal (infectious) transmission has been shown to be more prevalent in virulent rickettsiae species [27]. Multiple mechanisms for horizontal transmission have been elucidated for *R. felis* within cat fleas, as well as other invertebrate hosts. A prerequisite to successful horizontal transmission is oral acquisition of *R. felis*. This was demonstrated by an experiment exposing uninfected cat fleas to an *R. felis*-infected bloodmeal in an artificial host system, where cat fleas were shown not only to be able to acquire the infection, but to also remain persistently infected for up to 28 days post-exposure [22]. Demonstration of transmission through a shared blood meal was confirmed via an experiment where cat fleas—both infected and uninfected—fed on an artificial host. Uninfected cat fleas became infected with *R. felis* at varying rates (3.3–40.0%), as early as 24-h post-exposure to infected fleas [26]. In this experiment, it was also shown that cat fleas could become infected through mating with infected cat fleas without exposure to any infectious bloodmeal. Co-feeding is the successful horizontal transmission of pathogens between actively blood-feeding arthropods in the absence of a disseminated vertebrate infection, and has also been demonstrated in cat fleas infected with *R. felis*. Infected (donor) cat fleas were placed in either the same capsules (co-fed) or different capsules (cross-fed) as uninfected (recipient) cat fleas on an uninfected murine host. Recipient cat fleas were shown to acquire the infection in both co-fed and cross-fed models in the absence of the murine hosts becoming rickettsemic. Interestingly, the experiment also revealed that infected cat fleas were able to transmit *R. felis* to naïve rat fleas (*Xenopsylla cheopis*) [3].

There has been further investigation into the specific mechanism of horizontal transmission of *R. felis* between cat fleas. Support for salivary transmission was found when *R. felis* was detected via qPCR in the salivary gland of cat fleas that had been feeding on cats for 2–4 days [28]. Definitive evidence for salivary gland localization within cat fleas was given when rickettsial organisms were visualized via IFA in salivary glands in previously uninfected cat fleas 7–14 days post-exposure to an infectious blood meal [29]. Given these findings, as well as the previously discussed studies on co-feeding, there is strong evidence for *R. felis* transmission through infectious saliva in cat fleas (e.g., biological transmission). Recent evidence for mechanical transmission has also been demonstrated in cat fleas. Previously uninfected cat fleas were shown to be infectious to naïve cat fleas as early as 24 h post-exposure to an infectious blood meal, indicating early-phase transmission. In addition, *R. felis* could not be detected in the salivary glands of these infectious cat fleas and the organism was shown to be released from contaminated mouthparts during probing [30].

While *R. felis* is primarily transmitted by *C. felis*, multiple field studies have demonstrated molecular detection of the infectious agent in not only other species of fleas, but also ticks, mites, and mosquitoes [13,20]. However, it is unclear whether these other arthropods contribute to the ecology of *R. felis*, or if their *R. felis*-infection is transient and insignificant in transmission [21]. Recently, ticks exposed to *R. felis* maintained rickettsiae for one generation, but transmission was not stable [31]. Likewise, *Anopheles gambiae* mosquitoes demonstrated the ability to sustain an infection for up to 15 days, but stable transmission was not observed [32]. Various genotypes of *R. felis* have also been isolated from several of these other non-flea arthropods [21], including a novel strain of *R. felis* that was identified in the non-blood-feeding booklouse, *Liposcelis bostrychophila* [33,34]. This strain of *R. felis* (str. LSU-Lb) has been shown to have the ability to not only infect cat fleas, but to undergo vertical transmission within these arthropods as well [25]. Genetic variation was not only found between strains isolated from different hosts (e.g., cat flea vs. booklouse), but also from strains isolated from the same host at different geographic locations [27]. Interestingly, *R. felis* seems to have a variable effect on the host, depending on the vector. As stated previously, vertical transmission of *R. felis* in *C. felis* has been highly variable, suggesting that this organism has a negative fitness effect on the arthropod population, requiring additional horizontal transmission for pathogen maintenance. However, in the booklouse, *R. felis* has been shown to be maintained 100% transovarially, and clearance of the organism from adults actually resulted in decreased longevity, fecundity, and non-viable egg production [33,35]. Given the variation reported within arthropods, further work investigating the effect of strain variation within various arthropod species must still be done.

## 5. Transmission to Vertebrates

While several transmission mechanisms within arthropods have been described in relation to infection with *R. felis*, the means by which vertebrates acquire an infection from these arthropods remains unclear. Transmission of flea-borne pathogens is often multifactorial, with each species having several transmission routes to ensure maintenance [30]. The most common route of flea-borne pathogen transmission to vertebrates is through the bite of an infected arthropod. Evidence that this could be a possible infection route for *R. felis* in *C. felis* was given by demonstrating the organisms within the salivary glands of fleas [28,29]. Further, naïve cats exposed to cat fleas infected with *R. felis* seroconverted after four months, and *R. felis* DNA was detected via qPCR in the blood of 5/16 of these cats [36]. However, definitive culture of the organism from the blood of exposed cats could not be obtained. A survey study that sampled over 100 cats from several states in the United States found none to have detectable levels of *R. felis* in the blood; however, one cat did have detectable levels on the skin and another on the gingiva [37]. The cutaneous presence of *R. felis* in one feline patient, combined with the lack of circulating organisms, is reminiscent of the cutaneous presentation of human patients in Africa and the Yucatan, although no cutaneous lesions were reported in the cat.

Another possible mechanism for vertebrate infection is via infectious vector feces. Excretion of viable rickettsiae in feces of infected arthropods has been found to be crucial in the transmission for other species, including *R. prowazekii* and *R. typhi*. The most common form of exposure to infectious arthropod feces is through cutaneous inoculation, either through the deposition of arthropod feces at the bite site or contamination of broken skin or wounds with feces. Transmission of *R. prowazekii* has been demonstrated to occur via scarification of a louse bite site with rickettsiae-laden feces [38], and cutaneous inoculation of feces from fleas infected with *R. typhi* has been shown to create infections in rat and man (with as little as 0.2 mg of flea feces producing infection) [39,40]. A closely-related bacterial species, *Bartonella henselae*, has also been shown to use this transmission mechanism for dissemination to vertebrates. Feces from cat fleas infected with the bacterium caused cats to become bacteremic 1–2 weeks post-intradermal injection, and caused seroconversion by 20 weeks post-injection [41].

There is evidence to suggest that *R. felis* is also transmitted via infectious flea feces. Egg-free feces from *R. felis*-infected *C. felis* fleas was assessed at days 2–28 post-exposure to an infectious blood meal. *R. felis* gDNA was detected at most time points throughout the study via qPCR amplification of the 17-kDa gene. Additionally, there is evidence that these are viable, transcriptionally active rickettsial organisms, because an *R. felis* transcript was detected in the feces at 21 days post-exposure to an infectious bloodmeal [22]. Further work with vertebrates must be performed to determine if this is a possible *R. felis* transmission mechanism in vivo.

It is difficult to study transmission of *R. felis* to vertebrate hosts because a definitive host with appropriate clinical signs and bacteremia has not been found. Several animals, including cats, dogs, opossums, raccoons, rodents, and humans, have been found to be either seropositive or PCR-positive for *R. felis* DNA. Additionally, the cat flea lacks true host specificity, and *R. felis*-infected arthropods have been recovered from cats, dogs, rodents, opossum, hedgehogs, horses, sheep, goats, gerbils, and monkeys [20]. Given the lack of a definitive mammalian host, many research experiments have looked at the transmission of *R. felis* from cat fleas to vertebrates using rodents, including mice and rats. Information about the choice of mouse strain could be taken by previous experiments performed on related species, such as *R. parkeri*—a member of the SFG rickettsiae. Several strains of inbred mice, including A/J, BALB/c, C3H/HeJ, and C3H/HeN, were studied to determine their response to intravenous and intradermal inoculation of *R. parkeri* [42]. The only strain to show pathology consistent with sustained infection was C3H/HeN, which exhibited marked facial edema and splenomegaly, as well as characteristic eschar-like lesions. Given this information, the C3H/HeN strain was more extensively studied with regards to *R. felis* infection. After intravenous inoculation with a high-dose of *R. felis* (1 × 10^6^ organisms), C3H/Hen mice had detectable levels of rickettsial DNA in the spleen and liver as early as one day post-inoculation. Levels decreased to 50% or less by six and 14 days post-injection. *Rickettsia felis* DNA was never detected in the blood of the mice, and no mice exhibited any overt clinical signs of illness or pathology [Macaluso, unpublished data]. This mouse strain was also used in the previously described co-feeding experiment, where mice received an interdermal inoculation with 5 × 10^9^ rickettsiae from culture. Mice in this study also did not show any evidence of clinical signs or *R. felis* DNA in their blood, although other organs including liver and spleen were not tested for the presence of *R. felis* gDNA [3].

A recent study looked at the BALB/c mouse strain, in regards to the ability to acquire an *R. felis* infection. This study utilized mosquitoes, *Anopheles gambiae*, to examine the transmission to vertebrates. This approach was undertaken to further investigate the paradox of the low number of infected cat fleas found in areas of high *R. felis* prevalence in Africa. Natural bites from *R. felis*-infected *A. gambiae* mosquitoes were shown to produce a transient rickettsemia in BALB/c mice, confirmed via qPCR analysis of the blood [32]. The bacteremia was present in a majority of the mice both one and two days after being exposed to infected mosquitoes, but disappeared by day three. However, even though this mouse model was able to acquire an infection, no clinical signs or physical changes were reported in these mice. While several of these mouse models have shown some promise, it is apparent that a definitive laboratory model that mimics the clinical disease in humans has yet to be found.

## 6. Discussion

It is clear that there is still much to be understood about *R. felis*. While the cat flea is still believed to be the primary reservoir and vector of the organism worldwide, the discovery of multiple arthropods that harbor the pathogen reveals the need for more extensive field research, including analysis of all possible arthropods in the area of reported human disease. A more complete picture of the possible vectors of human disease could propel research in the right direction. In addition, given that multiple transmission mechanisms within *C. felis* have been elucidated, it is possible that the transmission of *R. felis* within and amongst other arthropods is equally as complicated and multifactorial. Experiments that include transmission of the organism to multiple vectors might more closely mimic what is happening in nature. The most perplexing question that remains to be answered is how humans acquire the infection. While bites from infected cat fleas were previously thought to be the most likely mechanism, multiple laboratory experiments have not been able to produce a rickettsemic vertebrate with clinical signs that mimic a human infection through this route. It is important to note that given the findings in Africa of non-rickettsemic patients that exhibit clinical signs, an appropriate laboratory model may also not show evidence of *R. felis* infection in the blood. Additionally, mammals may simply be asymptomatic reservoirs. Further research with vertebrates, such as association with other *R. felis*-infected arthropods or contact with infectious arthropod feces, might aid in answering these questions and discovering a definitive disease-causing transmission mechanism from arthropod to human.
